# Supplementary figures and images for: The Aedes aegypti peritrophic matrix controls arbovirus vector competence through HPx1, a heme–induced peroxidase
Source: PLoS Pathog. 2023 Feb 13;19(2):e1011149. doi: 10.1371/journal.ppat.1011149 (PMC9956595; doi:10.1371/journal.ppat.1011149)

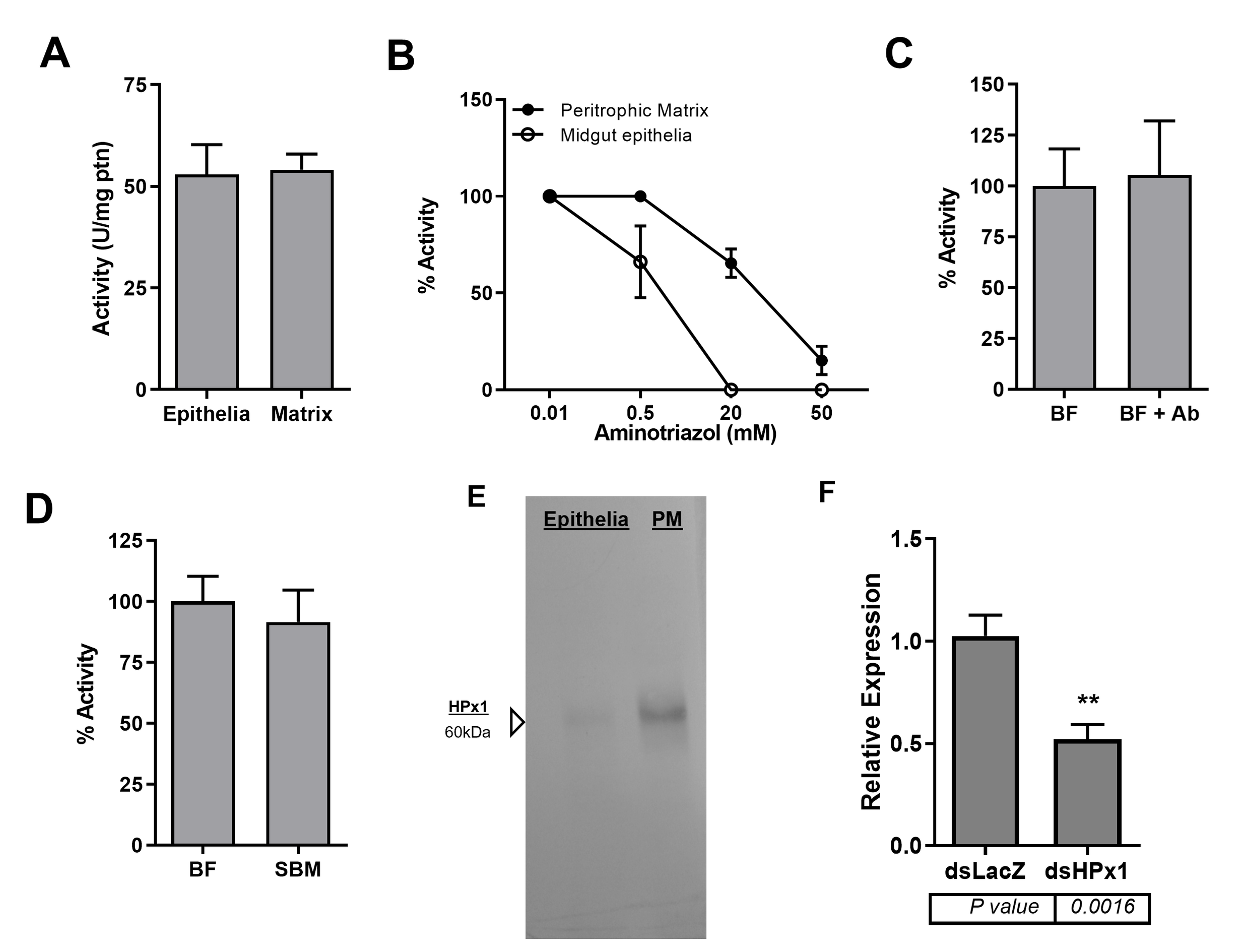

Supplement: S1 Fig — A) Catalase-specific activity comparison of the intestinal epithelia and PM at 24 h ABM. B) In vitro sensitivity of midgut epithelia and PM samples to aminotriazole, a catalase/peroxidase inhibitor. C) Mosquitos were treated with (or without) an antibiotic cocktail in a sugar meal, and PM catalase activity was assayed at 24 h ABM (BF n = 10; +AB n = 11). (D) Catalase activity comparison of the PM at 24 h ABM for mosquitoes fed blood or SBM, a chemically defined artificial diet (BF n = 21; +AB n = 17). E) HPx1 western blot of midgut epithelia and PM protein extracts, referring to Fig 1E. F) HPx1-silencing efficiency in the mosquito gut 24 h ABM. (TIF) [file ppat.1011149.s001.tif]

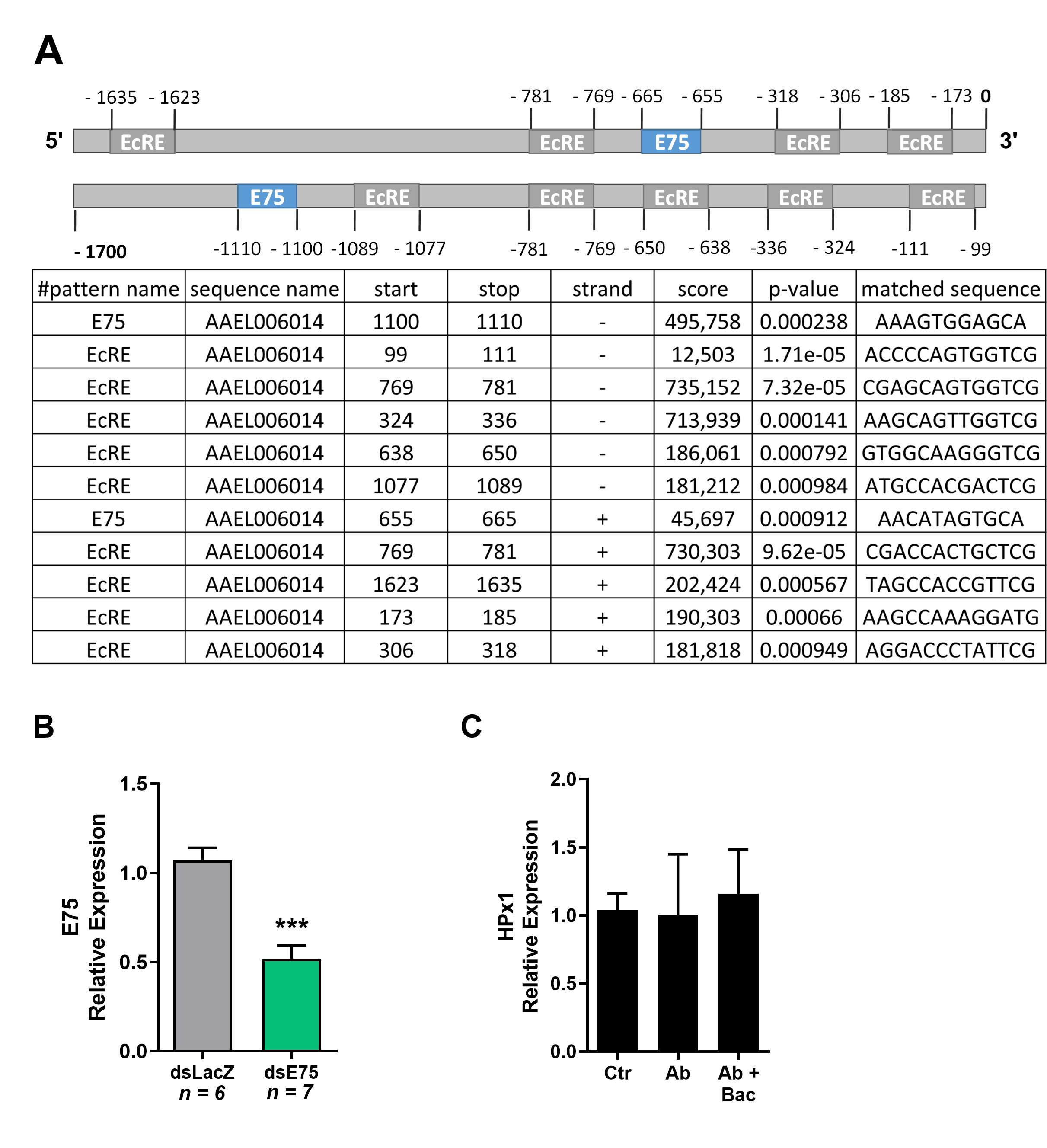

Supplement: S2 Fig — A) Schematic illustration of the E75 and ecdysone receptor-binding motifs location in the promoter region of HPx1. Numbers refer to nucleotide positions relative to the transcription start site. Table show output of the FIMO analysis including p-values and sequences for each putative TF binding site. B) E75 silencing efficiency in midguts at 24 h ABM. C) HPx1 expression in midguts at 24 h ABM. Control mosquitoes were fed a regular sucrose solution before blood feeding. Ab mosquitoes were pretreated with antibiotics before blood feeding. Ab + Bac mosquitoes were pretreated with antibiotics and fed blood containing Enterobacter cloacae at 1 OD/ml (Ctr n = 6; Ab n = 6; Ab+Bac n = 5). Data are the mean +/- SEM. (TIF) [file ppat.1011149.s002.tif]

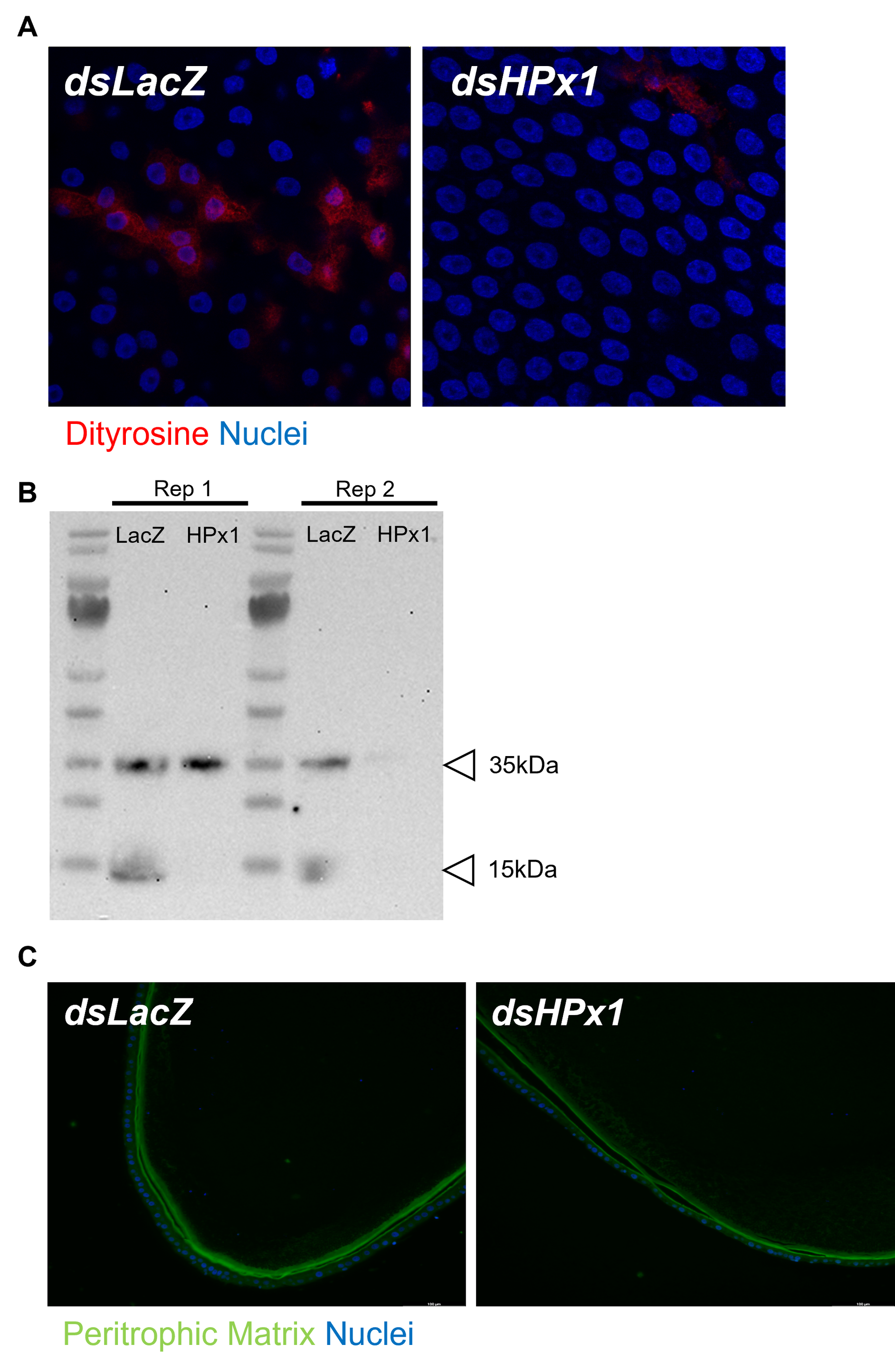

Supplement: S3 Fig — A) Dityrosine immune staining of mosquito midgut epithelia 18 h ABM. The protocol used was the same published by Kumar et al., 2010 [22] for IMPer characterization in Anopheles gambiae. B) Western blotting detection of dityrosine in protein extracts of PM pools of dsLacZ and dsHPx1. Briefly, 25μg of protein of each sample was resolved in an SDS gel and wet transferred to a PVDF membrane for 1h, 100V in cold room. The primary antibody was diluted in blocking solution (1:1000) ON at 4°C. The membrane was developed using HRP-SuperSignal West Dura Substrate (Invitrogen). C) Peritrophic matrix staining by WGA-FITC. Midguts were dissected 18 h ABM, fixed ON in 10% neutral buffered formalin solution at room temperature, paraffin-embedded, and sectioned. The WGA staining was performed as in Talyuli et al., 2015 [47]. (TIF) [file ppat.1011149.s003.tif]

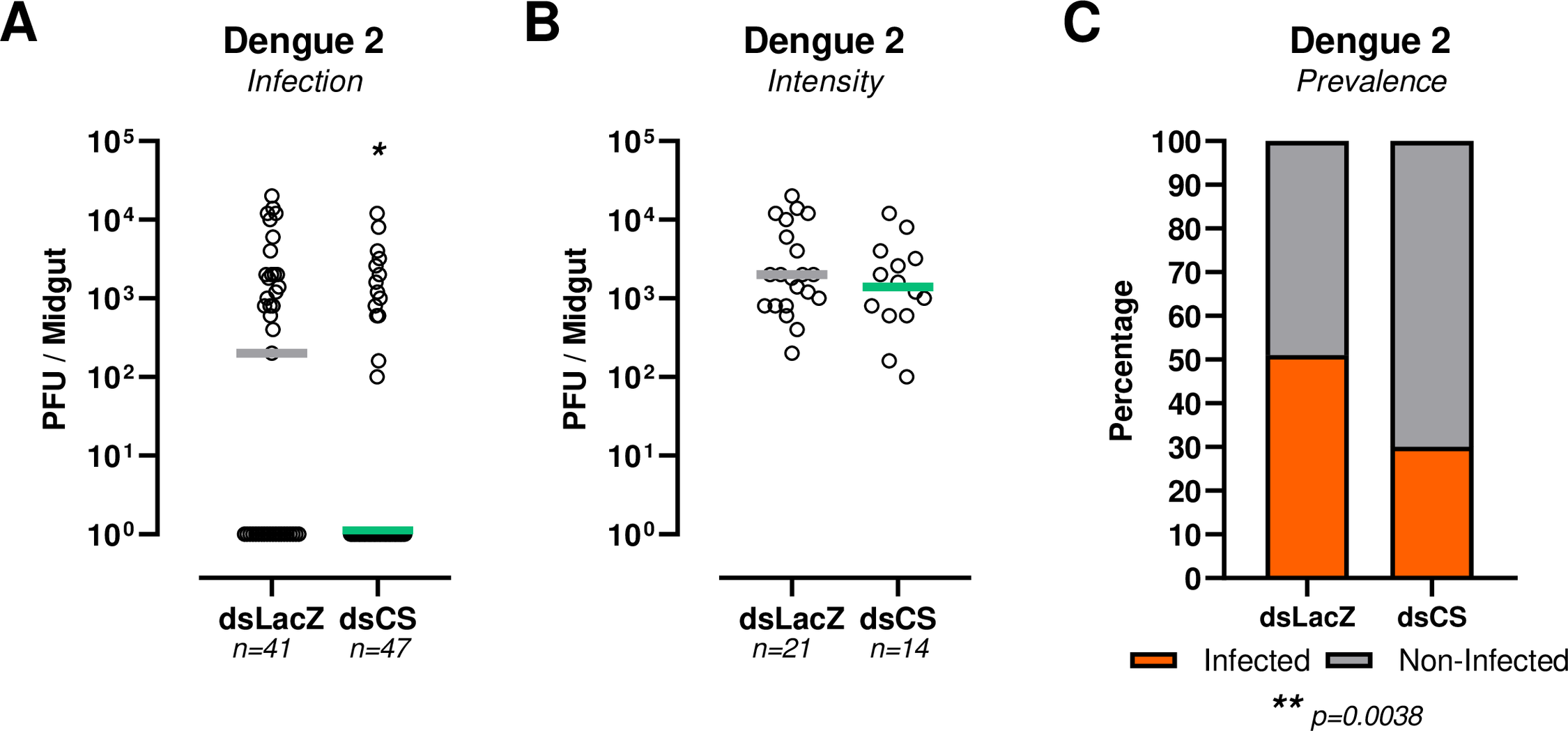

Supplement: S4 Fig — A) Dengue 2 titers in midguts 7 days postinfection. Viral titers were assessed by the plaque assay. Each dot represents an individual mosquito gut, and bars indicate the median. * p< 0.05; Mann–Whitney test. The prevalence statistical analysis was performed by the Chi-square test followed by Fisher’s exact test. (TIF) [file ppat.1011149.s004.tif]

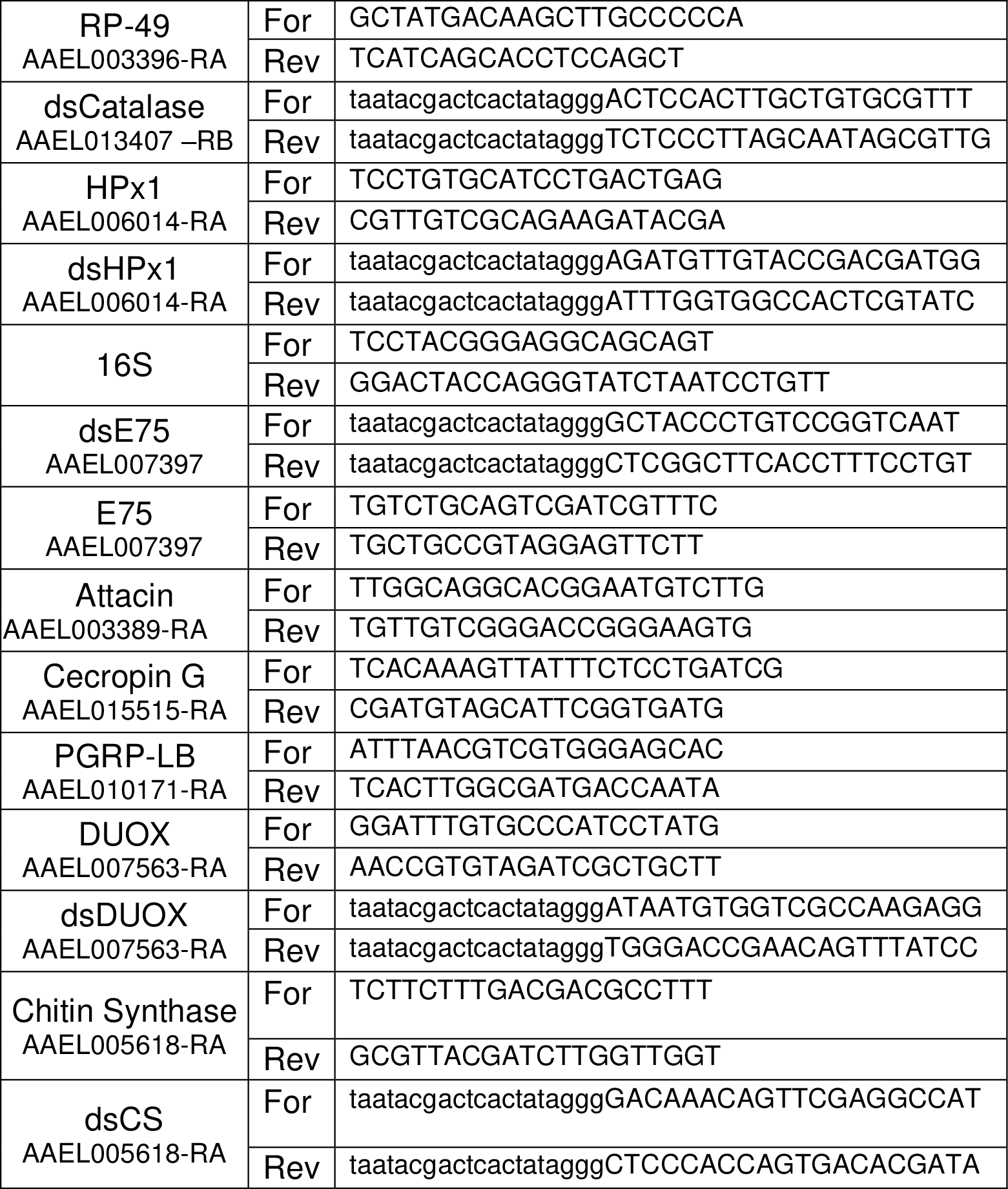

Supplement: S1 Table — (TIF) [file ppat.1011149.s005.tif]
